# Breast cancer subtypes and survival in patients with brain metastases

**DOI:** 10.1186/bcr1870

**Published:** 2008-02-28

**Authors:** Byung-Ho Nam, Sun Young Kim, Hye-Sook Han, Youngmee Kwon, Keun Seok Lee, Tae Hyun Kim, Jungsil Ro

**Affiliations:** 1 Center for Clinical Trials, National Cancer Center, 111 Jungbalsan-ro, Ilsandong-gu, Goyang-si, Gyeonggi-do 410-769, Republic of Korea; 2 Center for Breast Cancer, National Cancer Center, 111 Jungbalsan-ro, Ilsandong-gu, Goyang-si, Gyeonggi-do 410-769, Republic of Korea; 3 Center for Proton Therapy, National Cancer Center, 111 Jungbalsan-ro, Ilsandong-gu, Goyang-si, Gyeonggi-do 410-769, Republic of Korea

## Abstract

**Introduction:**

Brain metastases (BM) occur in up to one third of patients with metastatic breast cancer (MBC), whose incidences and prognoses by breast cancer subtypes in BM have not been well delineated.

**Methods:**

Retrospective survival analyses were performed in 126 BM patients from 805 MBC patients treated at the National Cancer Center between August 2001 and April 2006, according to clinical characteristics, breast cancer subtypes, and receipt of trastuzumab. Estrogen receptor (ER), progesterone receptor (PR), and human epidermal growth receptor-2 (HER2) statuses were tested by immunohistochemical (IHC) staining, and HER2 FISH analysis conducted for IHC 2+.

**Results:**

The proportion of HER2+/ER- (29% vs 16%) and triple-negative (37% vs 25%) tumors was higher in the 126 BM patients than those without BM. While median survival after recurrence was longer in patients with luminal A disease (median survival of luminal A vs luminal B vs HER2+/ER- vs triple-negative: p = 0.0246; 39.6 vs 27.4 vs 20.9 vs 15.5 months), survival was shorter from BM to death in luminal A and triple negatives (median survival: p = 0.0113; 4.0 vs 9.2 vs 5.0 vs 3.4 months). Receipt of trastuzumab after BM was a significant variable for survival in HER2+ patients. Multivariate analyses identified ER-negative, HER2-negative, or triple-negative, as well as older age, presence of leptomeningeal disease, and three or more extracranial disease sites, as poor prognostic factors for survival after BM.

**Conclusion:**

MBC patients who developed BM had higher proportions of triple-negative and HER2+/ER- tumor status. Triple receptor status is a useful prognostic marker for predicting survival after BM in metastatic breast cancer patients.

## Introduction

Brain metastases (BM) clinically present in up to 16% of all metastatic breast cancers (MBC), although autopsy series reveal approximately twice as many cases (34%) [1,2]. Of the patients with BM at autopsy, 19% are identified as having leptomeningeal disease (LMD) [1]. Prognosis of BM is extremely poor, and the median survival of BM patients ranges from 3 to 6 months [3], as it is usually a late event of systemic disease. The general medical condition is often deteriorated by progressive neurological disabilities. Among several risk factors identified in previous studies, negative estrogen receptor (ER) or progesterone receptor (PR) [4-8] and human epidermal growth factor receptor-2 (HER2) overexpression [6,9] have been associated with a higher risk of development of BM.

Recent gene expression studies using DNA microarrays have revealed the prognostic implication of breast cancer intrinsic subtypes, including the basal-like, HER2+/ER-, and two types of ER+ tumors: luminal A and luminal B subtypes [10,11]. Immunohistochemical (IHC) surrogates of these subtypes that were determined by IHC profiles of ER, PR, HER2, HER1 and cytokeratin 5/6 were also studied. Although the definition does not exactly coincide with that of gene-array-defined intrinsic subtype, and only 30% to 50% of array-defined luminal B tumors are HER2+, the surrogate IHC subtypes were also shown to be equally correlated with patient survival [12].

Based on the premise that the outcome of BM patients is affected by the specific breast cancer intrinsic subtype, we conducted the current study to analyze the prognostic significance of clinical and biologic characteristics in correlation with IHC tumor subtypes in BM patients with breast cancer.

## Materials and methods

### Study population

Between August 2001 and April 2006, a total of 805 metastatic breast cancer patients were evaluated at the National Cancer Center Hospital, Goyang, Korea. They were initially followed up to October 2006. Of the 805 patients, 138 (17.1%) presented symptomatic BM during their disease course. BM was diagnosed by imaging studies with magnetic resonance imaging or computed tomography and/or lumbar punctures. Screening by brain imaging in asymptomatic patients is not performed routinely at the National Cancer Center. The triple receptor (ER, PR and HER2) status was known for 682 of the 805 metastatic breast cancer patients, including 126 of the 138 patients with BM. Another group of 118 serial patients, who were diagnosed with early breast cancer (EBC) between January 2002 and April 2003 with no evidence of recurrence until October 2006, was screened for HER2 by fluorescence *in situ *hybridization (FISH) and analyzed for their subtype status. The proportion of breast cancer subtypes was compared among three different disease subsets; patients with EBC (*n *= 118), MBC without BM (*n *= 556), and MBC with BM (*n *= 126).

Patients with BM were followed up further to July 2007. The medical records were retrospectively reviewed for the clinical data, including age at the initial diagnosis and at BM, initial stage of the disease, pathologic type, number and site of extracranial metastases, disease-free interval, number of brain metastatic lesions, presence of leptomeningeal disease, treatment of BM, and survival from the onset of metastasis and from the diagnosis of BM. This study protocol was approved by the Institutional Review Board of the National Cancer Center (IRB protocol number NCCNCS 07-093). Because this study was a retrospective analysis that involved no more than minimal risk for the subjects, the institutional review board approved our request for the waiver of informed consent.

### Immunohistochemistry and fluorescence *in situ *hybridization

IHC staining was performed on tissue sections cut from formalin-fixed, paraffin-embedded representative breast tumors. Staining was performed with the I-View DAB detection kit and Ventana ES autostainer (Ventana Medical Systems, Tucson, AZ, USA), using primary antibodies against ER (Ventana Medical Systems), PR (Ventana Medical Systems) and a polyclonal anti-HER2 oncoprotein (Herceptest; DAKO, Carpinteris, CA, USA). For the evaluation of ER and PR expression, we selected any focal positivity as weakly positive expression [13]. For HER2 expression, only membranous staining was scored according to HercepTest (DAKO) protocol criteria [14].

FISH analysis was performed mostly on IHC 2+ tumors using the PathVysion HER2 DNA Probe Kit (Vysis, Downers Grove, IL, USA). HER2-positive staining was defined as IHC 3+ or, in the case of IHC 2+, FISH positive. HER2-negativity was defined as IHC 0, 1+ or 2+, along with negative FISH results. If IHC staining or HER2-FISH was not available in the case of IHC 2+, HER2 status was categorized as unknown. Due to unknown ER, PR, or HER2 status, 12 out of 138 breast cancer patients with BM were excluded for further analysis. HER2 status was examined by FISH analysis for all the tumor samples of the 118 EBC patients, and HER2 was considered positive upon detection of HER2 amplification.

We divided the patients into four different breast cancer subtypes: ER or PR+ and HER2-, ER or PR+ and HER2+, ER- and PR- and HER2+, and ER- and PR- and HER2-. These subtypes were referred to as luminal A, luminal B, HER2+/ER-, and the triple-negative phenotype, respectively. The triple-negative phenotype was characterized by absence of staining for ER, PR, and HER2 receptors, similar to the basal-like subtype, which has added characteristics of positive staining for basal-cell (myoepithelial) cytokeratins (CKs) 5/6 and/or HER1+ [12]. As we have not performed IHC for CK 5/6 and/or HER1, all cases of ER-, PR- and HER2- were classified as showing a triple-negative phenotype.

### Treatment of brain metastases

Patients with BM were treated as indicated with at least one of the following treatment modalities: whole brain radiotherapy (WBRT), stereotactic radiotherapy, metastasectomy, or intrathecal chemotherapy (IT). WBRT was performed at a dose of 30 Gy (grays) in 10 fractions using a 6-MV photon by two lateral opposed standard fields covering all intracranial contents. Stereotactic radiotherapy was delivered with a 6-MV photon coupled to a micro-multileaf collimator (3-mm width); patients were immobilized with the fixation pins in a stereotactic frame or a thermoplastic stereotactic head mask (Brain LAB AG, Heimstetten, Germany). Tumors with a maximum diameter of 3 cm or less were treated with dose of 15–22 Gy with a single fraction, while tumors larger than 3 cm were treated with dose of 36 Gy in 6 fractions, 5 times each week. IT for patients with leptomeningeal disease was performed via lumbar puncture or Ommaya reservoir with methotrexate (10–15 mg twice a week) until malignant cells were cleared in the cerebrospinal fluid.

### Statistical analyses

A chi-square test was performed to compare the incidence of each breast cancer subtype in the three different patient subsets: patients with EBC, MBC without BM, or MBC with BM. Chi-square and Fisher's exact test were used for categorical variables to compare the patient characteristics among the four subtypes in 126 BM patients whose triple receptor statuses were known. A Kruskall-Wallis test was performed to compare the median intervals among these four groups. The overall survival from recurrence to death and from BM to death was estimated using the Kaplan-Meier method and compared using the log-rank test. The univariate and multivariate Cox regression analyses were used to identify the independent predictive factors that significantly influenced the overall survival from BM to death. All the statistical analyses were performed using the STATA software, Version 9 (StataCorp, College Station, TX, USA).

## Results

### Breast cancer subtypes according to disease status

The proportion of each IHC subtype according to the extent of disease is shown in Table 1. The median follow times up to October 2006 were 45.1 months for EBC group, 31.1 months for MBC without BM group, and 26.2 months for BM group. Each disease subset contained a different proportion of IHC subtypes (p < 0.0001). While more than half of the EBC patients were luminal A, the proportion of patients with HER2+/ER- or triple-negative tumors was significantly higher in the BM population compared to the EBC population, suggesting that these subtypes were associated with the development of BM. Notably, the proportion of luminal B tumors did not change from the early stage to BM.

**Table 1:** Breast cancer subtypes according to disease status.

IHC subtype	No. (%)			p Value†
		
	Patients with EBC (*n *= 118)	Patients with MBC* (*n *= 556)	Patients with BM (*n *= 126)	
Luminal A (ER or PR+/HER2-)	68 (57.6)	254 (46.7)	23 (18.3)	< 0.0001
Luminal B (ER or PR+/HER2+)	16 (13.6)	73 (13.1)	19 (15.1)	
HER2+/ER- (ER-/PR-/HER2+)	15 (12.7)	91 (16.4)	37 (29.4)	
Triple-negative (ER-/PR-/HER2-)	19 (16.1)	138 (24.8)	47 (37.3)	

Abbreviations: BM, brain metastases; EBC, early breast cancer; ER, estrogen receptor; HER2, human epidermal growth factor receptor-2; IHC, immunohistochemistry; MBC, metastatic breast cancer; PR, progesterone receptor.

*Excluding patients with brain metastases.

†Comparing three groups (patients with EBC, patients with MBC other than BM, and patients with BM) using the chi-square test.

### Clinical and tumor characteristics of patients with BM

The clinical features of the 126 patients with BM and the relationship with receptor status are presented in Table 2. In all, 23 tumors (18.3%) were luminal A tumors, 19 (15.1%) were luminal B, 37 (29.4%) were HER2+/ER-, and 47 (37.3%) were triple-negatives. Bone metastases were more frequent in luminal A or B tumors compared with others subsets, while other clinical characteristics were not significantly different among the four subtypes.

**Table 2:** Clinical characteristics of patients with BM.

	No. (%)					p Value*
		
	Total (*n *= 126)	Luminal A (*n *= 23)	Luminal B (*n *= 19)	HER2+/ER- (*n *= 37)	Triple-negative (*n *= 47)	
Age, mean (range), y	47 (22–70)	46 (34–65)	48 (29–70)	48 (28–69)	47 (22–69)	0.773

Age at BM						
≤ 45 years	59 (46.8)	13 (56.5)	8 (42.1)	16 (43.2)	22 (46.8)	0.746
> 45 years	67 (53.2)	10 (43.5)	11 (57.9)	21 (56.8)	25 (53.2)	

AJCC stage:						0.812
I	20 (15.9)	3 (13.0)	1 (5.3)	6 (16.2)	10 (21.3)	
II	34 (27.0)	6 (26.1)	7 (36.8)	8 (21.6)	13 (27.7)	
III	41 (32.5)	7 (30.4)	6 (31.6)	14 (37.8)	14 (29.8)	
IV	22 (17.5)	4 (17.4)	5 (26.3)	7 (18.9)	6 (12.8)	
Unknown	9 (7.1)	3 (13.0)	0 (0.0)	2 (5.4)	4 (8.5)	

Histology:						0.863
IDC	115 (91.3)	20 (87.0)	18 (94.7)	33 (89.0)	44 (93.6)	
ILC	6 (4.8)	1 (4.3)	1 (5.3)	2 (5.4)	2 (4.3)	
Mucinous	1 (0.8)	1 (4.3)	0 (0.0)	0 (0.0)	0 (0.0)	
Metaplatic	4 (3.2)	1 (4.3)	0 (0.0)	2(5.4)	1 (2.1)	

Disease-free interval, median (IQR), months	19.0 (25.0)	31.8(35.0)	26.9 (36.9)	17.5 (20.0)	17.0 (20.7)	0.1075

Interval from recurrence to BM, median (IQR), months	11.5 (15.2)	17.5 (36.0)	12.2 (17.0)	11.2 (14.3)	11.1 (14.5)	0.1473

Clinical features of BM:						0.053
Multiple	102 (81.0)	15 (65.2)	18 (94.7)	33 (89.2)	36 (76.6)	
Single	11 (8.7)	2 (8.7)	1 (5.3)	1 (2.7)	7 (14.9)	
LMD	13 (10.3)	6 (26.1)	0 (0.0)	3 (8.1)	4 (8.5)	

Number of disease sites:						0.834
< 3	42 (33.3)	9 (39.1)	5 (26.3)	13 (35.1)	15 (31.9)	
≥ 3	84 (67.7)	14 (60.9)	14 (73.7)	24 (64.9)	32 (68.1)	

Extracranical metastatic site:						
Bone	80 (63.5)	18 (78.3)	16 (84.2)	24 (64.9)	22 (46.8)	0.010
Lung/pleura	92 (73.0)	15 (65.2)	15 (78.9)	26 (70.3)	36 (76.6)	0.683
Liver	52 (41.3)	10 (43.5)	13 (68.4)	14 (37.8)	15 (31.9)	0.053
Skin/soft tissue/LN	94 (74.6)	15 (65.2)	12 (63.2)	30 (81.1)	37 (78.7)	0.122

Abbreviations: AJCC, American Joint Committee on Cancer; BM, brain metastases; ER, estrogen receptor; HER2, human epidermal growth factor receptor-2; IDC, invasive ductal carcinoma; ILC, invasive lobular carcinoma; IQR, interquartile range; LMD; leptomeningeal disease; LN, lymph node.

*Comparing four groups (luminal A, luminal B, HER2+, triple-negative) using analysis of variances to test for differences in means, Kruskall-Wallis test for difference in medians, and chi-square or Fisher's exact test for the remaining characteristics.

Of the 126 patients with BM, 103 patients were treated with WBRT, 3 with stereotactic radiotherapy only, 6 with IT only, 9 received WBRT with IT, 2 with metastatectomy (op) followed by radiotherapy, and 2 with op only as initial therapy. One patient chose symptomatic therapy only. Of the 56 HER2-positive patients, 10 patients did not receive trastuzumab, 25 completed trastuzumab therapy prior to BM diagnosis, and 21 patients received trastuzumab continuously before and after the onset of BM, or only after BM diagnosed. No patients received trastuzumab in the adjuvant setting.

### Clinical outcome according to breast cancer subtypes

**Figure 1:**
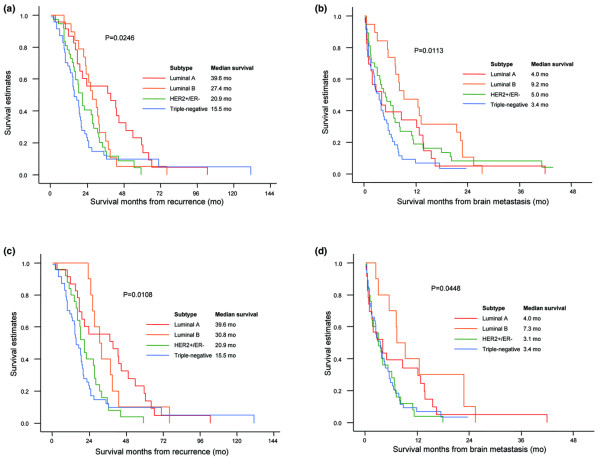
Kaplan-Meier survival curves according to triple receptor status **(a) **from recurrence to death of all patients (*n *= 126), **(b) **from brain metastasis to death of all patients (*n *= 126), **(c) **from recurrence to death, excluding patients who received trastuzumab after BM (*n *= 105), and **(d) **from brain metastasis to death, excluding patients who received trastuzumab after BM (*n *= 105).

As of July 2007, 120 of 126 patients were deceased, and the median age of all patients was 47 years (range, 22–70 years). With a median follow-up of 56.0 months from recurrence, the median survival of all patients from the time of recurrence to death was 20.0 months (range, 0.1–131.0 months). The median survival from recurrence varied depending on disease subset, with patients with luminal A at 39.6 months, luminal B at 27.4 months, HER2+/ER- at 20.9 months, and triple-negative tumors at 15.5 months (p = 0.0246, Figure 1a). With a potential median follow-up of 34.8 months from brain metastasis, the median survival for all patients was 4.5 months (range, 0.1–43.9 months) from the brain metastasis to death. Patients with ER+ or PR+ tumors lived significantly longer after BM compared with ER- and PR- tumors (7.3 vs 3.8 months, p = 0.0439), as did patients with HER2-positive tumors compared with HER2-negative tumors (6.7 vs 3.4 months, p = 0.0085). Survival after BM also significantly differed according to triple receptor status as determined by IHC and/or FISH. Patients with luminal A (4.0 months) and triple-negative tumors (3.4 months) had a similar shorter survival time compared with luminal B (9.2 months) and HER2+/ER- (5.0 months) tumors (p = 0.0113), as shown in Figure 1b.

**Figure 2:**
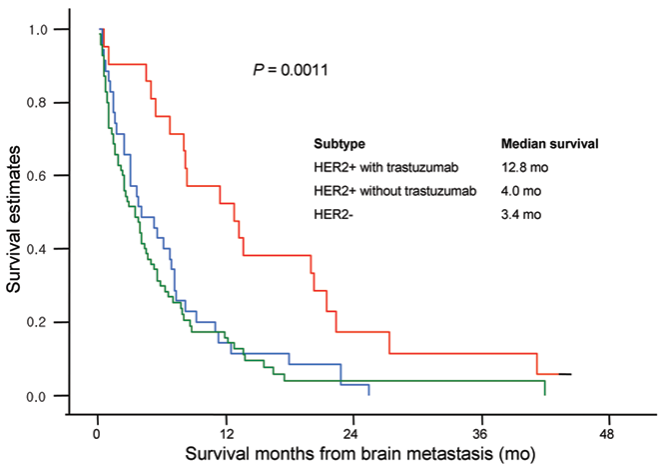
Kaplan-Meier curves of survival from brain metastasis according to HER2 status and receipt of trastuzumab.

Among the 56 HER2-positive patients, 9 of 19 patients with ER or PR+ tumors and 12 of 37 patients with ER- and PR- tumors received trastuzumab therapy after diagnosis of BM. When survival analysis was performed excluding those 21 patients who received trastuzumab after the onset of BM to eliminate the effect of trastuzumab on survival (Figure 1c, d), both survival from recurrence and survival from BM still significantly differed according to breast cancer subtypes. A small but significant survival gain in patients with luminal B (9.2 months vs 7.3 months) and HER2+/ER- (5.0 months vs 3.1 months) tumors upon delivery of trastuzumab therapy was observed (Figure 1b, d). Moreover, a significant survival benefit was noted in HER2-positive patients treated with trastuzumab after BM was diagnosed when all 126 patients were compared in the following three groups: 21 HER2-positive patients who received trastuzumab after the onset of BM (12.8 months) vs 35 HER2-positive patients who did not receive trastuzumab after the onset of BM (4.0 months) vs 70 HER2-negative patients (3.4 months) (p = 0.0011, Figure 2). Although patients were not randomized between those who received trastuzumab and those who did not receive trastuzumab in this retrospective analysis, patient characteristics such as number of brain metastases, extracranial metastatic sites, and performance status were balanced between the two groups of HER2+ patients.

### Cox regression analysis of clinical predictors

Univariate Cox regression analysis revealed a difference in survival related to hormone receptor or HER2 status. Survival after BM was significantly poorer in triple-negatives when compared with other types. Significant factors for survival after BM also included age at the onset of BM (≥ 45 yrs vs < 45 yrs), the disease free interval (< 24 months vs ≥ 24 months), the interval from the recurrence to BM (< 12 months vs ≥ 12 months), number of metastatic sites (≥ 3 vs < 3), and leptomeningeal disease (presence vs absence) in univariate analysis. When these significant variables for survival after BM were included in the multivariate Cox regression model, ER- and PR-, HER2-negative, triple-negative, age ≥ 45 yrs, ≥ 3 metastatic sites, and presence of leptomeningeal disease were each significantly associated with poor survival after the onset of BM (Table 3).

**Table 3:** Results of Cox regression analysis of survival predictors in 126 patients with BM.

Variable	Univariate analysis		Multivariate analysis	
	
	HR (95% CI)	p Value	HR (95% CI)	p Value
ER**- **and PR**- **vs ER or PR+	1.477 (1.006–2.168)	0.047	1.656 (1.107–2.478)	0.014*
HER2**- **vs HER2+	1.625 (1.125–2.347)	0.010	1.608 (1.099–2.352)	0.014*
Triple negative vs others	1.758 (1.194–2.588)	0.004	1.692 (1.137–2.518)	0.010*
Age ≥ 45 yrs vs < 45 yrs	1.815 (1.245–2.647)	0.002	1.700 (1.155–2.504)	0.007†
Disease free interval: < 24 months vs ≥ 24 months	1.857 (1.258–2.740)	0.002	1.379 (0.897–2.119)	0.143†
Interval from recurrence to BM: < 12 months vs ≥ 12 months	0.688 (0.478–0.988)	0.043	0.702 (0.478–1.031)	0.071†
Number of metastatic sites: ≥ 3 vs < 3	1.710 (1.155–2.532)	0.007	1.769 (1.141–2.743)	0.011†
Leptomeningeal disease: presence vs absence	2.069 (1.153–3.713)	0.015	2.865 (1.506–5.450)	0.001†

Abbreviations: BM, brain metastasis; CI, confidence interval; ER, estrogen receptor; HER2, human epidermal growth factor receptor-2; HR, hazard ratio; PR, progesterone receptor.

*Variables regarding triple receptor status were separately analyzed adjusting for other variables (age, disease free interval, interval from recurrence to BM, number of metastatic sites, leptomeningeal disease).

†Each variable other than triple receptor status was adjusted with triple-negative vs others.

## Discussion

ER negativity, HER2 overexpression, high tumor grade and large tumor volume of extracranial metastatic disease have all been reported as risk factors for developing BM after breast cancer.

More recent studies have focused on the HER2-positive breast cancer patients, where nearly one third of patients who received trastuzumab developed brain metastasis [15,16]. We observed a significant proportion of specific breast cancer subtypes in our series of BM, as shown by 37% triple-negative tumors and 29% HER2+/ER- tumors, likely as a consequence of the aggressive nature of these subtypes. Considering the study population was not a single cohort of patients who were diagnosed with initial breast cancer in the same time period and were followed over time, it is possible that the three different groups may have been a selected population. With these unequal factors counted, our study supports the addition of the triple-negative subtype to the list of risk factors for developing BM. Compared to the other subtypes, the proportion of luminal B subtypes did not vary among the three different disease status (Table 1).

Approximately 10–15% of all breast cancer presents a triple-receptor-negative phenotype, consistent with observations from the current study of early breast cancer. This subgroup shares many clinical and pathological features with the so called 'basal-like' subgroup according to gene expression profile analysis, first described by Perou *et al*. [10]. Pathologic characteristics of basal-like breast tumors are low expression of HER2 and ER, high mitotic index, and high expression of genes which are characteristic of the basal epithelial cell layer of breast duct (myoepithelial cells), including expression of cytokeratins 5, 6, and 17 [17]. Patients with this subtype not only have a poor prognosis but tend to develop more visceral metastasis, including brain metastasis, as shown in the current study [17,18].

Once brain metastasis occurs, the outcome for breast cancer patients is generally poor. Although radiotherapy or surgery has been used to improve the survival and quality of life of patients with BM, the outcome has not been generally satisfactory. The 1-year survival rate of breast cancer patients with BM is 20% [19], which is somewhat better than other types of cancer owing to the relatively indolent nature of the disease. Conventional prognostic markers affecting survival of BM patients include age, performance status and systemic tumor control, etc. Here, we studied the correlation between breast cancer subtypes as well as other clinical features and the survival of breast cancer patients with BM, and the results indicate that aside from the patient's age, number of extracranial disease sites and LMD, the triple receptor status affected survival from the recurrence as well as after the development of BM.

The prognostic implication of an intrinsic subtype or its IHC surrogate has been previously described in early breast cancer patients [10-12]. Carey *et al*. reported on the prevalence of IHC subtypes in correlation with survival in 469 early stage breast cancer patients in the pre-trastuzumab era with a minimum of 8.1 years of follow-up; in this study, the shortest survival was observed in patients with HER2+/ER- tumors (52%) in contrast to 75% in the basal-like subtype, 84% in luminal A, and 87% in luminal B [12]. Not only could the breast cancer subtypes predict the disease recurrence and survival of EBC patients, but such subtypes could also be used for predicting the outcome of patients with BM as in the current study. It must be noted that the triple-negative subtype defined in our study contains both array-defined basal-like tumor and unclassified subtypes, since we have not performed immunohistochemical staining for HER1 and cytokeratin 5/6 [12]. Survival rates from recurrence to death and from BM to death were significantly different among breast cancer subtypes, which are strongly rooted on the intrinsic differences in tumor biology among breast cancer subtypes. In the current study, BM with triple-negative tumors was associated with the gravest prognosis among all the subtypes.

In the multivariate analysis, not only were the conventional prognostic factors, including age, number of extracranial metastatic sites, and presence of LMD, associated with the survival from the time of BM, but also both the ER and HER2 status affected survival. The survival in patients with triple-negative disease was significantly shorter from disease recurrence to death compared with those with luminal A tumors (15.5 months vs 39.6 months, p = 0.0225). The overall survival characterized by prolonged disease-free interval and longer survival from the recurrence to death in patients with luminal A tumors could be explained by the indolent nature of the disease as well as the responsiveness to hormonal agents. However, the survival time was as low as for triple-negatives once the tumors in luminal A patients metastasized to the brain (4 months vs 3.4 months, p = 0.166). This short survival is most likely due to the lack of further therapy since BM presents in the very late phase of the disease in the patients with luminal A tumors. By contrast, the poor prognosis of triple-negative tumor is likely due to its intrinsic biological aggressiveness as well as the paucity of subsequent treatment to control the systemic disease [17]. The present study suggests that the patients with triple-negative tumors and HER2+/ER- tumors may have a higher risk to develop BM than the hormone receptor-positive subgroup, and no survival difference was observed between triple-negative tumors and HER2+/ER- tumors after excluding patients who received trastuzumab after BM diagnosis (3.4 months vs 3.1 months, p = 0.9253). However, patients with HER2-positive disease who received trastuzumab lived longer (12.8 months vs 4.0 months, p = 0.0019). Consequently, triple-negative tumors have become the worst prognostic group since the emergence of trastuzumab. Although trastuzumab does not cross the blood-brain barrier and has no direct activity on brain metastases, several studies have shown a survival benefit with trastuzumab in HER2-positive patients with BM, who had a significantly longer survival time compared with HER2-negative patients [20,21]. This improvement of survival could be largely attributed to the effects of trastuzumab in controlling systemic metastasis. However, it must be noted that potential selection biases dependent on the different clinical and social circumstances for the population in this retrospective study may have existed in prescribing trastuzumab.

## Conclusion

In summary, breast cancer patients with triple-negative and HER2+/ER- tumors appear to have a high risk for developing brain metastasis. Breast cancer subtypes, age of the patient, number of metastatic sites, and leptomeningeal disease were significantly correlated with survival after the onset of BM. Equally short survival times were observed in patients with luminal A and triple-negative tumors compared with luminal B and HER2+/ER- tumors after the onset of brain metastasis. Most likely the survival time gain by the latter two HER2-positive groups was due to the use of trastuzumab therapy, although there seemed to be an intrinsic biological difference for survival in these two groups. Together, our results show that ER, PR and HER2 combined receptor status could be useful in predicting survival even after BM diagnosis. Further studies are required to develop strategies to cover issues such as screening, as well as management for patients with triple-negative subtype BM.

## Abbreviations

BM = brain metastasis; ER = estrogen receptor; FISH = fluorescence *in situ *hybridization; HER2 = human epidermal growth factor receptor-2; IHC = immunohistochemistry; LMD = leptomeningeal disease; MBC = metastatic breast cancer; PR = progesterone receptor.

## Competing interests

The authors declare that they have no competing interests.

## Authors' contributions

BHN and JR designed the study. SYK, THK and JR conducted the data acquisition. BHN performed the statistical analysis. BHN, SYK, YK, THK and JR drafted the manuscript. BHN, SYK and JR revised the manuscript. BHN, SYK and JR participated in the interpretation of data. BHN, SYK, HSH, KSL and JR participated in critical review of the manuscript. All authors read and approved the final manuscript.
